# Supplementary material for: Unveiling the immunomodulatory properties of starch microparticles on alveolar macrophages
Source: PLoS One. 2025 Jul 3;20(7):e0327718. doi: 10.1371/journal.pone.0327718 (PMC12225817; doi:10.1371/journal.pone.0327718)
Supplement: S1 File — (DOCX) [file pone.0327718.s001.docx]

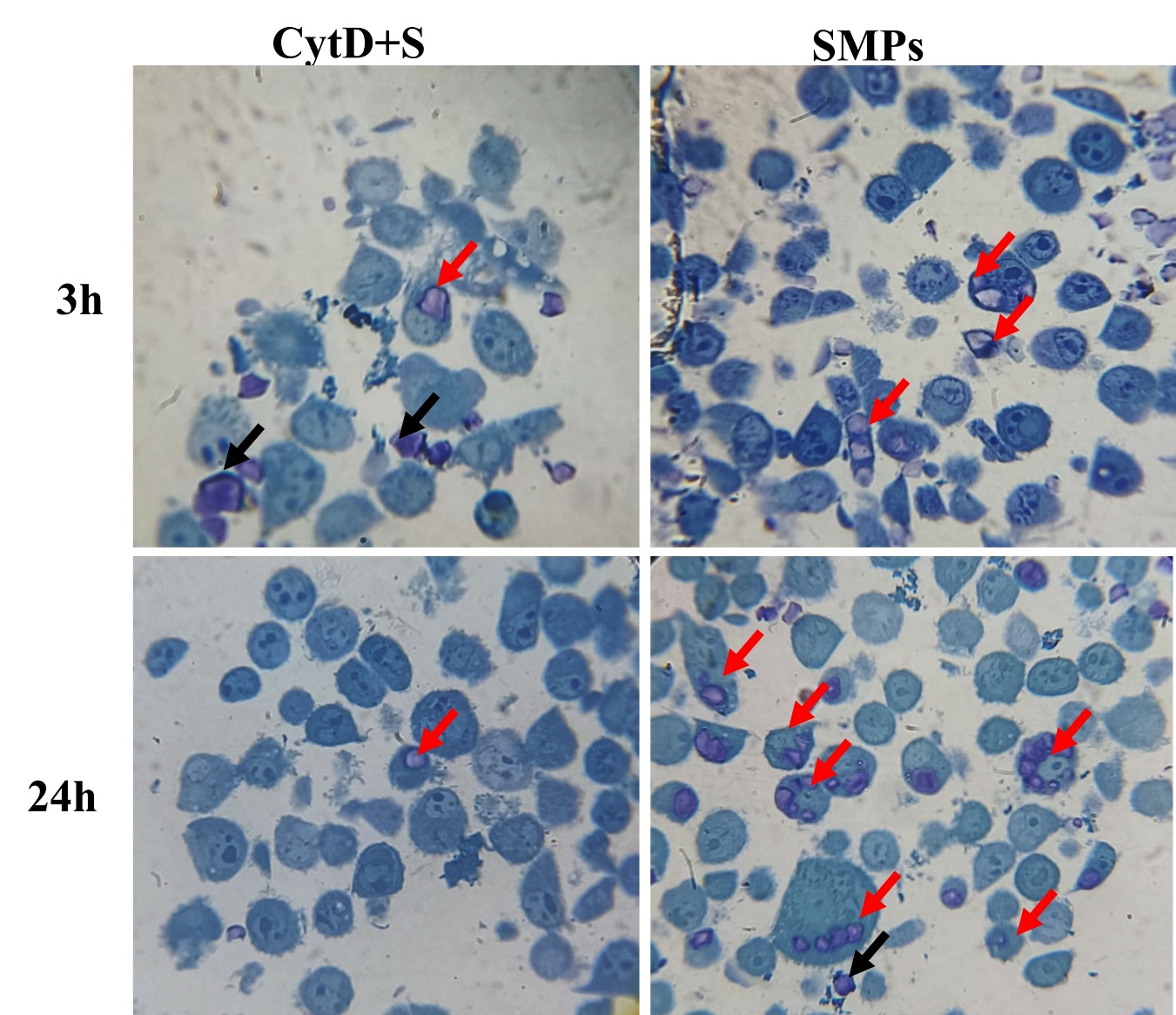


**S1 Fig. Semithin sections (0.5-1 μm) stained with toluidine blue**. MH-S macrophages with and without SMPs under a light microscope. In the left panels, most of the SMPs observed are outside of the cells (black arrows). In the right panels, most of the SMPs observed are inside the cells (red arrows).


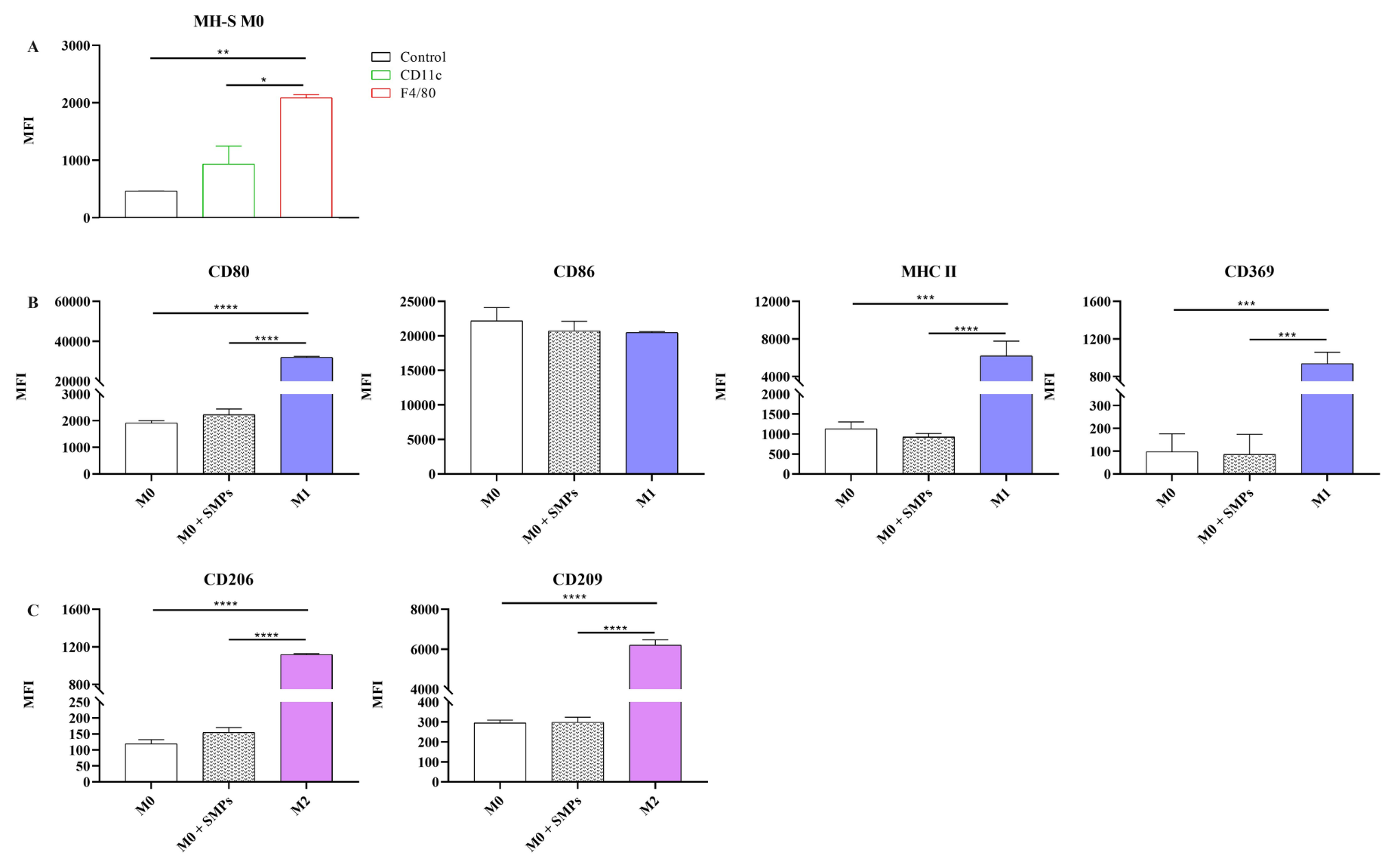


**S2 Fig.** **Expression of activation markers of MH-S alveolar macrophages stimulated with SMPs.** Bars represent the median fluorescence intensity (MFI) ± SEM of two independent experiments (n=6). **A)** Linage markers on M0 macrophages without stimulation. Cells without staining are shown as controls. **B)** Expression of M1 markers on M0 macrophages with and without SMPs and on macrophages polarized with LPS+INF-γ (blue bars). **C)** Expression of M2 markers on M0 macrophages with and without SMPs and on macrophages polarized with IL-4+IL-13 (pink bars). Two-way ANOVA and One-way ANOVA and Tukey’s multiple comparisons ***p<0.001, ****p<0.0001).
