# Supplementary material for: Unveiling the immunomodulatory properties of starch microparticles on alveolar macrophages
Source: PLoS One. 2025 Jul 3;20(7):e0327718. doi: 10.1371/journal.pone.0327718 (PMC12225817; doi:10.1371/journal.pone.0327718)
Supplement: Attachment — Submitted filename: Response to Reviewers.docx [file pone.0327718.s004.docx]

**Journal Requirements:**

1. When submitting your revision, we need you to address these additional requirements.

Please ensure that your manuscript meets PLOS ONE's style requirements, including those for file naming.

After revising the PLOS ONE style templates, we made the corresponding changes to meet requirements. The changes appear in the 'Revised Manuscript with Track Changes' file and are applied in the “Manuscript” file.

2. Thank you for stating in your Funding Statement:

According to the guide for authors in the Financial Disclosure Statement section, all the funding or sources of support received during this study are entered in the submission form section and were eliminated from the manuscript file.

The new amended statement includes specific grant numbers, initials of authors who received each award, full names of entities that funded the study or authors, and the statement: “There was no additional external funding received for this study.” As follows:

A1-S-14446, SMM, Ciencia Básica Fondo Sectorial de la Secretaría de Educación Pública y el Consejo Nacional de Humanidades, Ciencias y Tecnologías (SEP-CONAHCyT)

A1-S-9849, RRS, Ciencia Básica Fondo Sectorial de la Secretaría de Educación Pública y el Consejo Nacional de Humanidades, Ciencias y Tecnologías (SEP-CONAHCyT)

IN 216722, RRS, Programa de Apoyo a Proyectos de Investigación e Innovación Tecnológica/Dirección General Asuntos del Personal/Universidad Nacional Autónoma de México (PAPIIIT/DGAPA/UNAM)

894774, ABR, Doctoral Scholarship Consejo Nacional de Humanidades, Ciencias y Tecnologías (CONAHCyT)

There was no additional external funding received for this study.

3. When completing the data availability statement of the submission form, you indicated that you will make your data available on acceptance. We strongly recommend all authors decide on a data sharing plan before acceptance, as the process can be lengthy and hold up publication timelines. Please note that, though access restrictions are acceptable now, your entire data will need to be made freely accessible if your manuscript is accepted for publication. This policy applies to all data except where public deposition would breach compliance with the protocol approved by your research ethics board. If you are unable to adhere to our open data policy, please kindly revise your statement to explain your reasoning and we will seek the editor's input on an exemption. Please be assured that, once you have provided your new statement, the assessment of your exemption will not hold up the peer review process.

As previously declared, yes, all data are fully available without restriction and the statement is completed in the submission form. All data underlying the reported findings are provided as part of the submitted manuscript or supporting information files. Our experimental data do not need to be deposited in public-specific repositories because they are not microarray data or gene sequences.

4. We notice that your supplementary figures are uploaded with the file type 'Figure'. Please amend the file type to 'Supporting Information'. Please ensure that each Supporting Information file has a legend listed in the manuscript after the references list.

The amended files were uploaded as supporting information, and the legends were listed in the manuscript after the references list.

5. Please review your reference list to ensure that it is complete and correct. If you have cited papers that have been retracted, please include the rationale for doing so in the manuscript text, or remove these references and replace them with relevant current references. Any changes to the reference list should be mentioned in the rebuttal letter that accompanies your revised manuscript. If you need to cite a retracted article, indicate the article’s retracted status in the References list and also include a citation and full reference for the retraction notice.

The reference list was carefully revised and edited to ensure that it is complete and correct. No retracted papers have been cited. Editions appear in the 'Revised Manuscript with Track Changes' file.

Reviewers' comments:

Reviewer's Responses to Questions

**Comments to the Author**

**Reviewer #1:** 

{1.} following sentence { lines 120-121} is not clear. Authors can check and correct it.
the crystals of formazan were dissolved with SDS and the lecture was made at 570 nm.

The sentence was edited and appears in lines 124 and 125 of the “Manuscript” file and is indicated in lines 129 and 130 of the 'Revised Manuscript with Track Changes' file.

All figures were processed with PACE before submission to meet requirements.
